# Hard-wired Epimysial Recordings from Normal and Reinnervated Muscle Using a Bone-anchored Device

**DOI:** 10.1097/GOX.0000000000002391

**Published:** 2019-09-23

**Authors:** Henry T. Lancashire, Yazan Al Ajam, Robert P. Dowling, Catherine J. Pendegrass, Gordon W. Blunn

**Affiliations:** From the * Department of Medical Physics and Biomedical Engineering, University College London, London, UK; † Research Department of Orthopaedics and Musculoskeletal Science, University College London, London, UK; ‡ Royal Free Hospital, London, UK; § School of Pharmacy and Biomedical Sciences, University of Portsmouth, Portsmouth, UK.

## Abstract

A combined approach for prosthetic attachment and control using a transcutaneous bone-anchored device and implanted muscle electrodes can improve function for upper-limb amputees. The bone-anchor provides a transcutaneous feed-through for muscle signal recording. This approach can be combined with targeted muscle reinnervation (TMR) to further improve myoelectric control.

**Methods:**

A bone-anchored device was implanted trans-tibially in n = 8 sheep with a bipolar recording electrode secured epimysially to the peroneus tertius muscle. TMR was carried out in a single animal: the peroneus tertius was deinnervated and the distal portion of the transected nerve to the peroneus muscle was coapted to a transected nerve branch previously supplying the tibialis anterior muscle. For 12 weeks (TMR) or 19 weeks (standard procedure), epimysial muscle signals were recorded while animals walked at 2 km·h^−1^.

**Results:**

After 19 weeks implantation following standard procedure, epimysial recording signal-to-noise ratio (SNR) was 18.7 dB (± 6.4 dB, 95% CI) with typical recordings falling in the range 10–25 dB. Recoveries in gait and muscle signals were coincident 6 weeks post-TMR; initial muscle activity was identifiable 3 weeks post-TMR though with low signal amplitude and signal-to-noise ratio compared with normal muscle recordings.

**Conclusions:**

Following recovery, muscle signals were recorded reliably over 19 weeks following implantation. In this study, targeted reinnervation was successful in parallel with bone-anchor implantation, with recovery identified 6 weeks after surgery.

## Abstract 2

Supplemental Digital Content is available in the text.

## INTRODUCTION

Current upper-limb amputation prostheses have 2 key limitations: attachment to the amputation stump and intuitive, reliable prosthesis control. Present attachment solutions, including sleeves and harnesses, are sources of discomfort or pain for users, causing poor transpiration and pressure sores.^1–3^ Users also prioritize prosthesis function, including dexterous control and sensory feedback.^1,2,4,5^

One approach to improving prosthesis attachment is transcutaneous bone-anchors.^6–8^ Bone-anchors allow direct skeletal attachment of prostheses: the bone-anchor stem is implanted within the medulla of the residual bone and exits through the skin distally on the residual limb. Implant designs necessitate a 1-stage^9,10^ or 2-stage^11–13^ surgical procedure, and approaches to sealing the skin interface and avoiding ascending infections vary, including skin-to-bone healing^12^ or the formation of a skin–implant interface.^9,14^

Commercially available active upper-limb prostheses provide body-powered or myoelectric control. Myoelectric control strategies, including pattern recognition and proportional regression approaches, can improve prosthesis function over on/off control with finite state machines for changing grip.^15^ Myoelectric control using skin surface electrodes has associated challenges. Signals vary due to changes in electrode location, skin conductivity due to perspiration, muscle movement relative to the skin surface, and limb shape changes. Avoiding skin surface electrodes will reduce complexity and discomfort for prosthesis users. Implantable systems, with electrodes on the muscle surface or within the muscle, can overcome these issues providing improved EMG (electromyography) quality and reduced variability.^16–23^ Transcutaneous bone-anchors can be used to create a hard-wired connection to implanted electrodes,^17,23,24^ avoiding wireless signal transmission and implanted electronics.

Surgical approaches can improve myoelectric control by directed reinnervation of residual muscles to amplify neural signals from nerves previously supplying the amputated limb.^25^ One approach: targeted muscle reinnervation (TMR) transfers upper-limb nerves to residual muscles of the torso or limb stump.^26–31^ An alternative, regenerative peripheral nerve interface, reinnervates free muscle transfers with transected residual nerves of the limb.^21,32–36^

This study assesses a combined approach for prosthetic attachment and control using a bone-anchored device and implanted epimysial electrodes. We have previously demonstrated this approach in a single animal over 12 weeks,^23^ and the present study validates this over an extended timescale with n = 6 animals. This study also investigates TMR alongside hard-wired epimysial electrodes in a single animal model, to observe recovery of function and signal quality over 12 weeks.

## METHODS

**Fig. 1.**
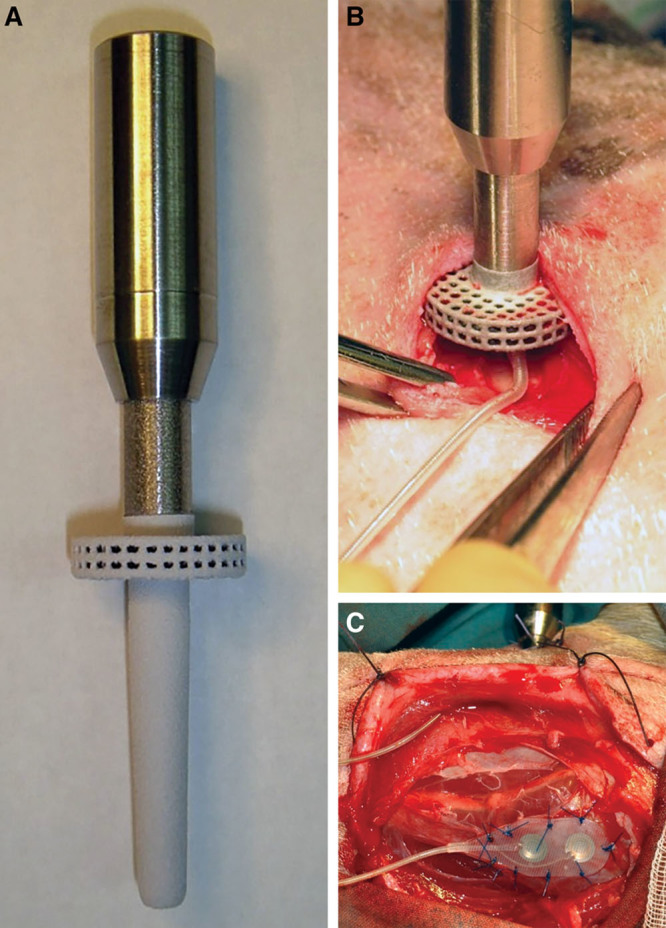
The bone-anchor and surgical procedure. A, The bone-anchor before epimysial array attachment. B, Bone-anchor inserted trans-tibially. C, Epimysial electrode array sutured to peroneus tertius muscle, with cable passing through a subcutaneous tunnel between the bone-anchor and the muscle.

The bone-anchored device is shown in Figure 1A. Devices were designed and manufactured as described previously.^23^ Transcutaneous bone-anchors with a porous flange were laser sintered from Ti-6Al-4V (Eurocoatings, Trentino, Italy)^37^ with 700 μm pore size and 300 μm strut size. Flange and tapered pin were hydroxyapatite plasma sprayed (Plasma Biotal, Tideswell, UK). A 2 mm diameter hole was drilled from the top-surface of the stem, exiting immediately below the porous flange.

Bipolar epimysial electrode arrays (Ardiem Medical Inc., Indiana, Pa.) with platinum–iridium electrodes were used (3.68 mm diameter, 10 mm center-to-center interelectrode distance). Arrays have reinforced backing and a 2-core, 316 stainless steel, coiled cable. A single array cable was passed through the drilled hole in each bone-anchor and connected to a 2-pin socket (LEMO UK Ltd., Worthing, UK). The socket was secured to the bone-anchor with a Ti-6Al-4V press-fit sleeve and epoxy resin, and voids were filled with medical grade silicone (MED3-4013, NuSil Technology LLC, Carpinteria, Calif.). Implants were sterilized using ethyl oxide gas.

### SURGICAL PROCEDURE

All in vivo procedures were carried out in compliance with The Animals (Scientific Procedures) Act UK, 1986 (revised 2013) and local guidance. All animals were skeletally mature female mule sheep. Sterile procedures were used throughout. A total of 8 animals were used for in vivo testing. In 7 animals, the bone-anchor and epimysial array were implanted following a previously described procedure.^23^ In one further animal, the bone-anchor and epimysial array were implanted and TMR was carried out.

#### BONE-ANCHOR IMPLANTATION

Anesthesia was induced with intravenous ketamine hydrochloride (Ketaset, Fort Dodge Animal Health Ltd., Sandwich, UK) and midazolam hypnovel (Roche Products Ltd., Welwyn Garden City, UK) and maintained with 2% isoflurane (IsoFlo) in oxygen. A 5 cm incision was made 10 cm inferior to the knee joint on the medial aspect overlying the left tibia. The bone-anchor was implanted trans-tibially: both cortices of the tibia were drilled, reamed, and the bone-anchor was press-fitted into the hole. A 2 mm gap between the flange and the tibia allowed the electrode cable to exit into the soft tissue (Fig. 1B).

#### ELECTRODE ARRAY IMPLANTATION

A 10 cm incision was made over the lateral compartment muscles of the left leg. The peroneus tertius muscle was identified.^38^ A subcutaneous tunnel was made between the lateral compartment incision and the bone-anchor incision, and the epimysial array was passed through the tunnel. The electrode was aligned with the long-axis of the muscle belly and sutured to the epimysium using 4-0 Prolene (nonabsorbable) suture (Fig. 1C).

#### TARGETED MUSCLE REINNERVATION

**Fig. 2.**
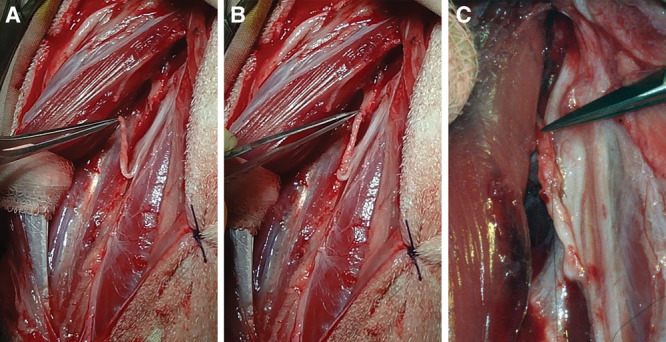
Targeted muscle reinnervation. A, Nerve branch to tibialis anterior transected. B, Nerve branch to tibialis anterior coapted to the motor nerve branch to the peroneus tertius muscle. C, Nerves sutured with 8-0 nylon filament.

The peroneous tertius muscle was exposed and retracted laterally to expose the peroneal nerve. Nerve branches to the tibialis anterior muscle and to the lateral compartment muscles were identified. One of 3 nerve branches to the tibialis anterior muscle was transected close to the muscle. The transected nerve branch was freed from surrounding tissue until the branch joined the peroneal nerve. The motor nerve branch to the peroneus tertius muscle was identified and transected 1 cm proximal to the insertion point. Epineurial repair was performed using three 8-0 nylon monofilament sutures (S&T, Neuhausen am Rheinfall, Switzerland) between the distal portion of the transected nerve to the peroneus muscle and the transected branch to the tibialis anterior muscle (Fig. 2A, B). The epimysial array was sutured onto the epimysium of the peroneus tertius muscle as above. The native motor branch to peroneus tertius muscle was cauterized to prevent spontaneous reinnervation.

#### CLOSURE AND POSTOPERATIVE MANAGEMENT

All incisions were closed in 2 subcutaneous and 1 cutaneous layers using Vicryl (absorbable) suture (Ethicon Inc., Somerville, N.J.). Wounds were dressed with Mepitel (Mölnlycke Health Care Limited, Bedfordshire, UK), sterile gauze, and bandage. Silicone masking caps (Greentree Shercon, Tewkesbury, UK) were used to protect the external sockets.

### ELECTROMYOGRAPHY

EMG was recorded during treadmill walking at 2 km·h^−1^. For the 7 animals which had not undergone TMR, recordings were made 1, 2, 3, 4, 6, 8, 14, and 19 weeks following implantation. For the single animal which had undergone TMR, recordings were made weekly for 12 weeks. The bone-anchor was connected using a shielded cable. A reference electrode was placed over a suitable bony prominence: the left-leg hock joint (ankle).

At 19 weeks, skin surface EMG recordings were made for comparison. The peroneus tertius muscle was identified by palpation. The skin was shaved and cleaned with alcohol. Ag-AgCl gel surface electrodes (11 mm electrode diameter; .Vermed Inc., Buffalo, N.Y.) were applied with an interelectrode distance of 20 mm.

Recordings were made using a BIOPAC EMG100C differential electromyogram amplifier and an MP150 data acquisition system with AcqKnowledge version 4.1.1 software (all from BIOPAC Systems, Inc., Goleta, Calif.). Recording parameters were: 1000 samples per second, 100–500 Hz band pass, 50 Hz notch filter, and 500× amplification.

Signal-to-noise ratio (SNR) was calculated for 6 gait cycles per recording according to Equation 1 using MATLAB 2017b (The MathWorks, Inc., Natick, MA, USA).^39^ Signal was identified visually; this was possible because at 2 km·h^−1^ 1 gait cycle occurs approximately each second. SNR was used as an estimate of signal quality.


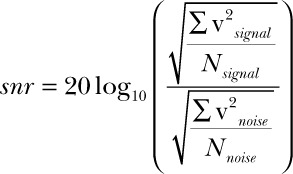
(1)

### PRETERMINAL MUSCLE STIMULATION

Crosstalk was assessed by preterminal stimulation of adjacent muscles in animals which had not undergone TMR. Under general anesthesia, the peroneus tertius, tibialis anterior, peroneus longus, and gastrocnemius muscles and their associated motor nerve branch(es) were identified and exposed by dissection. Motor nerves were stimulated using 1 mA pulses (Medtronic Vari-Stim III, Medtronic Inc., Fridley, Minn.). Six readings per muscle were recorded from the epimysial array on the peroneus tertius as described above. Following muscle stimulation, the animals were euthanized by intravenous injection of 0.7 mg/kg sodium pentobarbitone (Pharmasol Ltd., Hampshire, UK).

### FORCE PLATE ANALYSIS

For the TMR study, recovery of weight bearing was assessed using ground reaction force. Four days before surgery, and at regular intervals before or after EMG recording, the animal was walked across a force plate and at least 6 measurements for each hind limb (left and right) were recorded. Measurements were normalized for animal weight and reported as F_max_/weight.

### IMPEDANCE SPECTROSCOPY

Impedance spectroscopy was carried out as described previously^23^ from 10^3^ to 10^5^ Hz at 0.4 Vp-Hz at 0.4 Vp-p using an EVAL-AD5934EBZ impedance monitor (Analog Devices, Norwood, Mass.). Measurements were made before implantation, after 19 weeks in vivo under terminal anesthesia, and following explantation with 3 mm of surrounding tissue, in 0.9% saline solution.

### STATISTICAL ANALYSIS

Values are reported or plotted as mean ± 95% CI unless otherwise stated. Statistical comparisons were carried out using nonparametric tests using SPSS for Linux version 20 and SPSS for Windows version 22 (IBM Corp., Armonk, N.Y.).

## RESULTS

The single animal which had undergone TMR was euthanized after 12 weeks. A total of 5 animals were euthanized after 19 weeks. Two animals were euthanized before the intended endpoints. A single animal was euthanized at week 5 after diagnosis of a chronic infection of a surgical site for a different, concurrent study, and a single further animal was added to the study. A single animal was diagnosed with Johne’s disease, a paranatally acquired infection of ruminants not associated with this study^40^; this animal was euthanized at 12 weeks.

### GROSS MORPHOLOGY

Where bone-anchors were placed proximally on the tibia, skin movement caused sieving: an exposed flange with the epidermal layer within the flange, rather than above the flange. This complication was first observed at week 4 with thinning of the skin overlying the flange. In a single animal, the flange became visible at 8 weeks with dry necrosis of the skin, and no abnormal reddening, exudate, or other evidence of infection was observed. Where bone-anchors were placed in a more distal position, the skin–implant interface appeared stable. Fibrous capsules formed around the electrodes and cable in every animal overlying healthy muscle.

Following TMR, muscle atrophy was observed on the tibialis anterior and peroneus tertius muscles. The reinnervated nerve was in continuity, and no reinnervation was observed from the cauterized end of the native motor branch to peroneus tertius muscle (Fig. 2C).

**Fig. 3.**
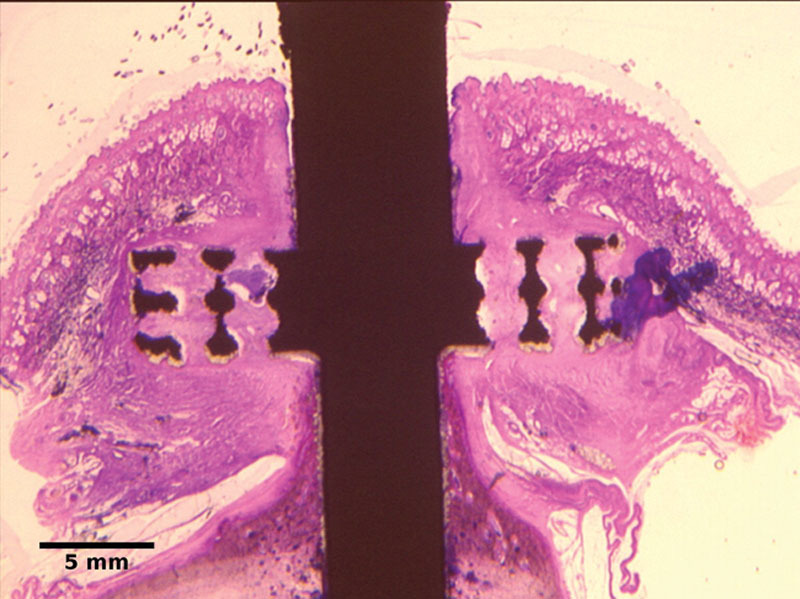
Skin–implant interface of a bone-anchored device, showing dermis integrated with the porous flange and some epidermal downgrowth. Reproduced with permission.^59^

Histological analysis was previously reported in Dowling (2015)^41^ and is summarized here. The explanted bone-anchors showed well-vascularized dermal tissue integration throughout the porous flange (Fig. 3) with 71 blood vessels per mm^2^ (median, 95% CI, 49–98 mm^−2^) infiltrating 90% of pores (median, 95% CI, 68%–100%). Where dermal tissue infiltration was poor, gaps at the tissue implant interface were observed. Downgrowth, where the epidermal cell layer descends along the implant shaft, was observed (5.36 mm median downgrowth from skin surface, 95% CI, 4.65–10.4 mm). Downgrowth through a thick skin section to the flange forms a sinus descending alongside the implant, and debris between the shaft and epidermis causes a pocket to form at the base of the sinus (see **figure, Supplemental Digital Content 1**, which displays epidermal downgrowth and sinus track formation along the implant shaft, **http://links.lww.com/PRSGO/B180**; and **figure, Supplemental Digital Content 2**, which displays debris impeding epidermal attachment at the base of a sinus, **http://links.lww.com/PRSGO/B181**).

Where downgrowth remained limited, contact between epidermal tissue and the bone-anchor shaft suggested a stable transcutaneous interface above the flange (see **figure, Supplemental Digital Content 3**, which displays epidermal attachment to the implant with limited downgrowth, **http://links.lww.com/PRSGO/B182**).

### EMG RECORDINGS

**Fig. 4.**
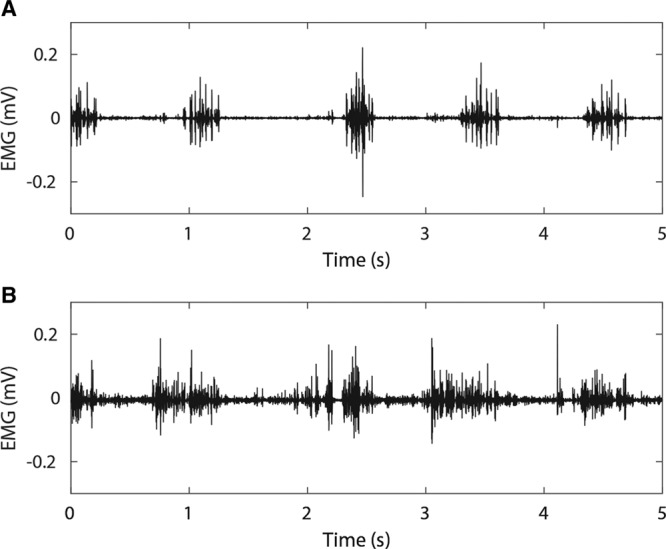
Raw EMG recordings from epimysial electrode array (A) and skin surface electrodes (B) after 19 weeks. Example traces recorded coincidentally from the same animal.

**Fig. 5.**
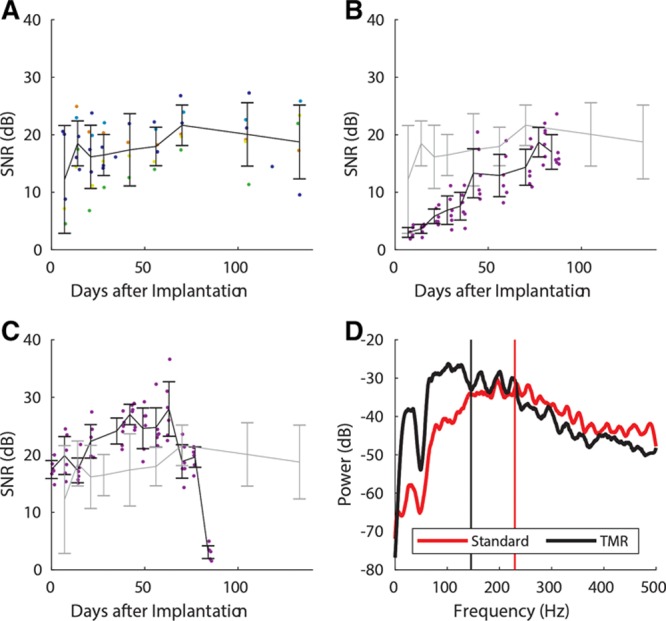
Signal changes following implantation. A, Summarized signal-to-noise ratio data from all animals with the standard surgical procedure. B, Signal-to-noise ratio following targeted muscle reinnervation (black), compared with standard procedure (gray). C, Signal-to-noise ratio from a previous single animal study 23 (black) compared with the standard procedure in the present study (gray). D, Example EMG power spectral density following targeted muscle reinnervation after 10 weeks (black, mean 145 Hz) compared with standard procedure after 19 weeks (red, mean 230 Hz). SNR data plotted as means ± 95% CI.

**Fig. 6.**
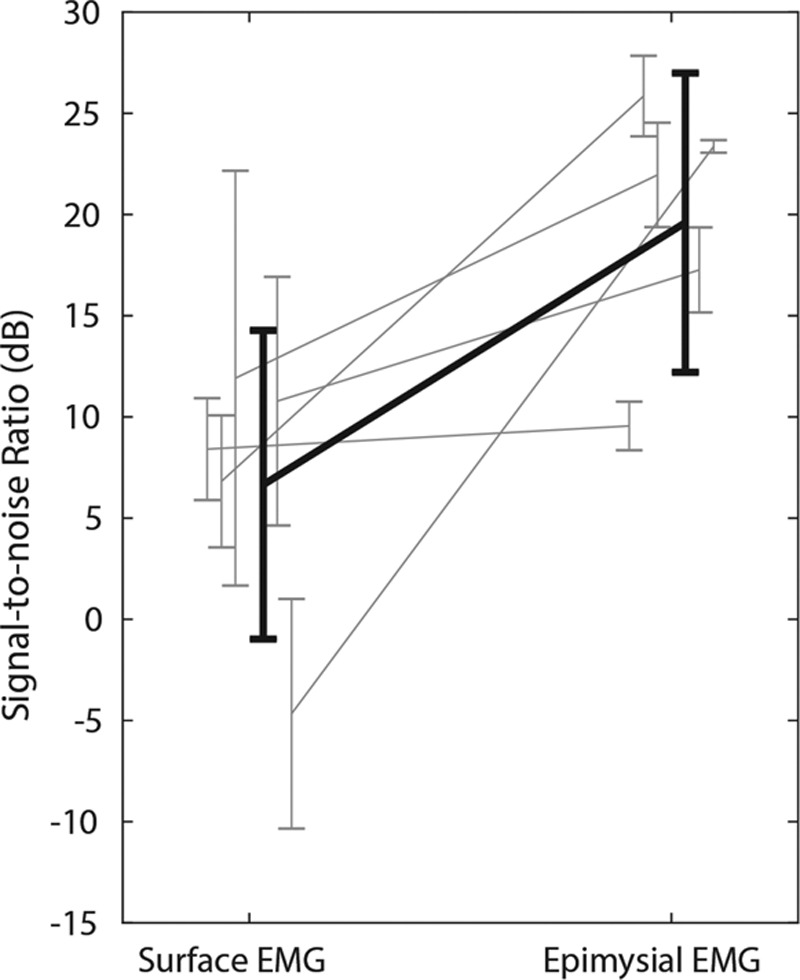
Slope chart of signal-to-noise ratio data at 19 weeks, comparing surface electrodes with epimyial electrodes. Black, combined data; Gray, individual animal data (n = 5). Data plotted as means ± 95% CI.

EMG recordings typically show low noise, with the gait cycle readily identifiable (see Fig. 4A and **figure, Supplemental Digital Content 4**, which displays Raw EMG and power spectrogram of EMG recordings from epimysial electrodes and skin surface electrodes after 19 weeks, **http://links.lww.com/PRSGO/B183**). Coincident surface electrode recordings show additional activity, increased noise, and less readily identifiable gait cycle (Fig. 4B and SDC4). SNR was typically in the range 10 to 25 dB and was 18.7 dB (± 6.4 dB, 95% CI) after 19 weeks (see Fig. 5A and **figure, Supplemental Digital Content 5**, which displays summarized SNR data for each individual animal with the standard surgical procedure, **http://links.lww.com/PRSGO/B184**). SNR agreed with previous work^23^ reassessed using the present analysis methods (Fig. 5C). At 19 weeks, SNR was greater for epimysial EMG (19.6 ± 7.4 dB) compared with surface EMG (6.65 ± 7.63 dB), but the difference was not statistically significant by Wilcoxon signed rank test (Z = 15, 7; *P* = 0.0625) (Fig. 6).

### EVOKED CROSSTALK

Crosstalk was assessed by stimulation of adjacent muscles under terminal anesthesia in animals which had not undergone TMR. Largest compound muscle action potentials (CMAPs) were observed when stimulating the peroneal nerve muscles: peroneus tertius (0.0338 ± 0.0032 V), peroneus longus (0.0200 ± 0.0019 V), and tibialis anterior (0.0077 ± 0.0010 V). Smallest CMAPs were observed when stimulating the 13 gastrocnemius (0.0048 ± 0.0012 V).

### EMG AND GAIT FOLLOWING TMR

**Fig. 7.**
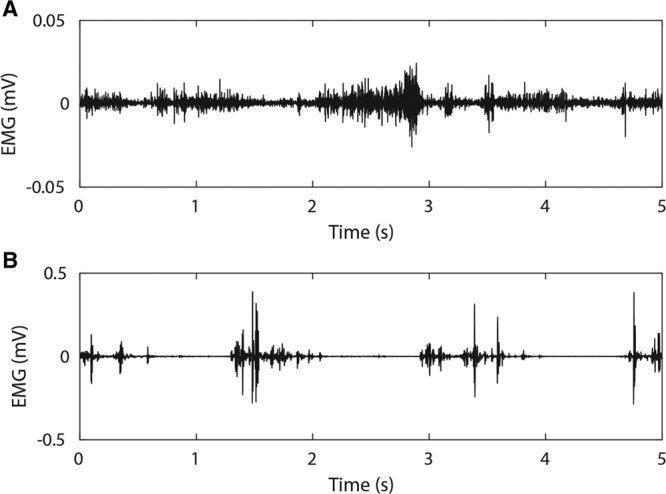
Raw EMG recordings following targeted muscle reinnervation. Recordings made at (A) 3 weeks and (B) 10 weeks. Note the change in voltage (y-)axis scale.

After 6 weeks following TMR, gait and EMG recovery had occurred. SNR was not different from recordings from animals without TMR after 6 weeks, but was lower before this time (Fig. 5B). Raw EMG shows some activity identifiable at 3 weeks (Fig. 7A), indicative of gait cycle; however, at low amplitude, it is not possible to differentiate crosstalk and reinnervation in this case. After 10 weeks, the gait cycle is readily identifiable (see Fig. 7B and **figure, Supplemental Digital Content 6**, which displays Raw EMG and power spectrogram of EMG recordings following TMR, **http://links.lww.com/PRSGO/B185**). Mean EMG frequency was lower following muscle reinnervation (Fig. 5D).

**Fig. 8.**
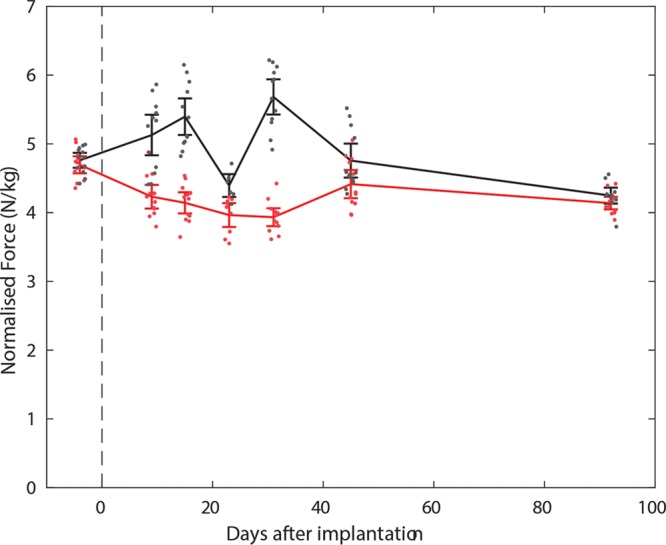
Change in normalized ground reaction force per leg following TMR on left leg; Right leg (black) and left leg (red). Dashed vertical line showing date of surgery.

After 6 weeks, weight bearing recovered such that normalized ground reaction force was not significantly different between legs (*P* ≥ 0.108, by Wilcoxon signed rank test) (Fig. 8). From 1 week after surgery to 4 weeks after surgery, inclusive ground reaction force was significantly reduced on the left (operated) leg (*P* ≤ 0.002, α_bonferroni_ = 0.007), except at week 3 where normalized ground reaction force was not significantly different (*P* = 0.064, by Wilcoxon signed rank test). This indicates that recovery of gait correlated with recovery of muscle function.

### ELECTRODE IMPEDANCE

Mean electrode impedance before implantation was 1.3 kΩ (1338 ± 17 Ω); after 19 weeks, in vivo mean impedance was 2.2 kΩ (2153 ± 388 Ω); and following explant and fixation in formal saline, mean impedance was 3.1 kΩ (3093 ± 2063 Ω).

## DISCUSSION

We have demonstrated epimysial EMG recording from 5 animals over 19 weeks, and a further 2 animals over 12 weeks and 1 animal over 5 weeks. The bone-anchored devices performed well, providing a transcutaneous cable route, while preserving the skin barrier. In a single animal, we observed EMG and functional recovery 6 weeks following TMR. The results presented in this paper further support the use of bone-anchors as hard-wired portals for biosignal recording.

The present study uses a trans-tibial insertion to minimize recovery time, allowing functional recordings from 1 week following implantation. In practice, bone-anchored devices are intended for longitudinal, intramedullary insertion following amputation, and similar devices, without the electrode cable, are in use clinically and in veterinary practice.^9,42^ Contrasting forces, for example, lack of weight bearing, therefore make this trans-tibial in vivo model less useful for assessment of the bone-implant interface. However, it remains valid to assess the skin–implant interface overlying and within the porous flange. The present flange is cylindrical, and alternative flange designs, including those in use clinically, are dome shaped^9,37^; we expect that a dome shape will reduce skin damage and sieving due to movement and pressure at the flange edge.

The implant design, with electrode cable(s) exiting directly into soft tissue below the porous flange presents advantages over other designs. Alternative bone-anchored portals have a cable exit within the bone medulla, requiring an additional surgical hole in the bone cortex for cable routing to the muscles.^17,24^ All designs introduce a path for infection along the cable within the bone-anchor. The present design is sealed with epoxy resin and medical grade silicone; however, for clinical use, a hermetic seal is preferable to restore the complete barrier within the bone-anchor.

This study assessed TMR in a single animal, showing functional, weight-bearing, recovery coincident with EMG recovery, 6 weeks after surgery. This study coapted a tibialis anterior nerve branch to the peroneus tertius motor nerve, which both act to extend the hock joint. Tibialis anterior function was not monitored; however, only 1 of 3 tibialis anterior motor nerve branches was transected to minimize functional impairment. Change in weight bearing may be due to the surgical procedure, rather than deinnervation and reinnervation, and the observed response may be due to healing and learning; a training effect may explain why EMG and weight-bearing recovery are coincident; and once the animal learns to use altered musculature, it can achieve a more normal gait. A control condition where the peroneus tertius motor nerve and single branch of the tibialis anterior nerve are transected and ligated to prevent reinnervation would allow differentiation between learning and reinnervation effects.

EMG recovery was assessed by SNR; however, subtler changes in reinnervated muscle occur including reduced EMG frequency, in agreement with previous investigation.^43^ This is possibly attributable to an increase in slow-twitch motor units, thereby reducing force output per motor unit. In self-reinnervated muscles, Ia afferent connections are lost, preventing proprioception, coordination, and stretch reflexes^44–46^; conflicting evidence from surgically reconstructed agonist–antagonist pairs has shown muscle spindle activity correlated with muscle stretch.^35^ Although TMR and regenerative peripheral nerve interface muscles no longer have roles in skeletal movement,^26^ maintaining proprioception would be beneficial.^47,48^ Reinnervation can also achieve sensory feedback, through targeted reinnervation of cutaneous nerves.^49,50^

Crosstalk is the portion of the EMG signal due to activation of nearby muscles, rather than the target muscle. Peak crosstalk CMAP decreased with increasing electrode–muscle distance.^51,52^ Reducing recording volume with smaller interelectrode distances (10 mm in this study) and tripolar electrode configurations can reduce crosstalk.^53–56^ The increased impedance in vivo supports previous studies showing a similar change.^16,23,51^

This study used bipolar epimysial electrode arrays limiting this study to one recording location. By combining reinnervated muscles with implanted multielectrode arrays, greater information about movement intention could be gained.^18^ We are developing an implantable EMG recording system,^57,58^ which can be combined with multielectrode arrays. The hard-wired bone-anchor approach is not limited to muscle recordings: it is suitable for neural recording where sampling rates ≥10 kHz per channel are required.

## ACKNOWLEDGMENTS

We would like to thank G. Hughes and R. Ferro de Godoy for technical assistance, and N. Donaldson and A. Vanhoestenberghe for helpful discussions.

## Supplementary Material

## References

[1] Cordella F Ciancio AL Sacchetti R Literature review on needs of upper limb prosthesis users. Front Neurosci. 2016;10:209. 27242413 10.3389/fnins.2016.00209 PMC4864250

[2] Benz HL Jia Y Rose L Upper extremity prosthesis user perspectives on unmet needs and innovative technology. Conf Proc IEEE Eng Med Biol Soc. 2016;2016:287–290. 28268333 10.1109/EMBC.2016.7590696 PMC5508653

[3] Dudkiewicz I Gabrielov R Seiv-Ner I Evaluation of prosthetic usage in upper limb amputees. Disabil Rehabil. 2004;26:60–63. 14660200 10.1080/09638280410001645094

[4] Kyberd PJ Hill W Survey of upper limb prosthesis users in Sweden, the United Kingdom and Canada. Prosthet Orthot Int. 2011;35:234–241. 21697204 10.1177/0309364611409099

[5] Biddiss E Beaton D Chau T Consumer design priorities for upper limb prosthetics. Disabil Rehabil Assist Technol. 2007;2:346–357. 19263565 10.1080/17483100701714733

[6] Pitkin M Design features of implants for direct skeletal attachment of limb prostheses. J Biomed Mater Res A. 2013;101:3339–3348. 23554122 10.1002/jbm.a.34606 PMC3758435

[7] Aschoff HH Transcutaneous osseointegration after limb amputation: A review over 27 years. Unfallchirurg. 2017;120:278–284. 28235982 10.1007/s00113-017-0329-y

[8] Hebert JS Rehani M Stiegelmar R Osseointegration for lower-limb amputation: a systematic review of clinical outcomes. JBJS Rev. 2017;5:e10. 10.2106/JBJS.RVW.17.00037 29087966

[9] Kang NV Pendegrass C Marks L Osseocutaneous integration of an intraosseous transcutaneous amputation prosthesis implant used for reconstruction of a transhumeral amputee: case report. J Hand Surg Am. 2010;35:1130–1134. 20541327 10.1016/j.jhsa.2010.03.037

[10] Al Muderis M Lu W Tetsworth K Single-stage osseointegrated reconstruction and rehabilitation of lower limb amputees: the osseointegration group of australia accelerated protocol-2 (OGAAP-2) for a prospective cohort study. BMJ Open. 2017;7:e013508. 10.1136/bmjopen-2016-013508 PMC5372148 28336738

[11] Al Muderis M Khemka A Lord SJ Safety of osseointegrated implants for transfemoral amputees: a two-center prospective cohort study. J Bone Joint Surg Am. 2016;98:900–909. 27252434 10.2106/JBJS.15.00808

[12] Li Y Brånemark R Osseointegrated prostheses for rehabilitation following amputation: the pioneering swedish model. Unfallchirurg. 2017;120:285–292. 28229193 10.1007/s00113-017-0331-4 PMC5371647

[13] Muderis MA Tetsworth K Khemka A The osseointegration group of australia accelerated protocol (OGAAP-1) for two-stage osseointegrated reconstruction of amputated limbs. Bone Joint J. 2016;98-B:952–960. 27365474 10.1302/0301-620X.98B7.37547

[14] Pendegrass CJ Goodship AE Blunn GW Development of a soft tissue seal around bone-anchored transcutaneous amputation prostheses. Biomaterials. 2006;27:4183–4191. 16618500 10.1016/j.biomaterials.2006.03.041

[15] Roche AD Rehbaum H Farina D Prosthetic myoelectric control strategies: a clinical perspective. Curr Surg Rep. 2014;2:1–11.

[16] Lewis S Russold M Dietl H Fully implantable multi-channel measurement system for acquisition of muscle activity. IEEE Trans Instrum Meas. 2013;62:1972–1981.

[17] Ortiz-Catalan M Håkansson B Brånemark R An osseointegrated human-machine gateway for long-term sensory feedback and motor control of artificial limbs. Sci Transl Med. 2014;6:257re6. 10.1126/scitranslmed.3008933 25298322

[18] Bergmeister KD Vujaklija I Muceli S Broadband prosthetic interfaces: combining nerve transfers and implantable multichannel EMG technology to decode spinal motor neuron activity. Front Neurosci. 2017;11:421. 28769755 10.3389/fnins.2017.00421 PMC5515902

[19] Bergmeister KD Hader M Lewis S Prosthesis control with an implantable multichannel wireless electromyography system for high-level amputees: a large-animal study. Plast Reconstr Surg. 2016;137:153–162. 26710019 10.1097/PRS.0000000000001926

[20] Pasquina PF Evangelista M Carvalho AJ First-in-man demonstration of a fully implanted myoelectric sensors system to control an advanced electromechanical prosthetic hand. J Neurosci Methods. 2015;244:85–93. 25102286 10.1016/j.jneumeth.2014.07.016 PMC4317373

[21] Vu PP Irwin ZT Bullard AJ Closed-loop continuous hand control via chronic recording of regenerative peripheral nerve interfaces. IEEE Trans Neural Syst Rehabil Eng. 2018;26:515–526. 29432117 10.1109/TNSRE.2017.2772961

[22] Morel P Ferrea E Taghizadeh-Sarshouri B Long-term decoding of movement force and direction with a wireless myoelectric implant. J Neural Eng. 2016;13:016002. 26643959 10.1088/1741-2560/13/1/016002

[23] Al-Ajam Y Lancashire H Pendegrass C The use of a bone-anchored device as a hard-wired conduit for transmitting EMG signals from implanted muscle electrodes. IEEE Trans Biomed Eng. 2013;60:1654–1659. 23358938 10.1109/TBME.2013.2241060

[24] Pitkin M Cassidy C Muppavarapu R Recording of electric signal passing through a pylon in direct skeletal attachment of leg prostheses with neuromuscular control. IEEE Trans Biomed Eng. 2012;59:1349–1353. 22345523 10.1109/TBME.2012.2187784 PMC3498509

[25] Hoffer JA Loeb GE Implantable electrical and mechanical interfaces with nerve and muscle. Ann Biomed Eng. 1980;8:351–360. 7027837 10.1007/BF02363438

[26] Kuiken TA Li G Lock BA Targeted muscle reinnervation for real-time myoelectric control of multifunction artificial arms. Jama. 2009;301:619–628. 19211469 10.1001/jama.2009.116 PMC3036162

[27] Kuiken TA Miller LA Lipschutz RD Targeted reinnervation for enhanced prosthetic arm function in a woman with a proximal amputation: a case study. Lancet. 2007;369:371–380. 17276777 10.1016/S0140-6736(07)60193-7

[28] Kuiken TA Feuser AES Barlow AK Targeted Muscle Reinnervation: A Neural Interface for Artificial Limbs. 2013 1st ed Boca Raton: CRC Press.

[29] Hijjawi JB Kuiken TA Lipschutz RD Improved myoelectric prosthesis control accomplished using multiple nerve transfers. Plast Reconstr Surg. 2006;118:1573–1578. 17102730 10.1097/01.prs.0000242487.62487.fb

[30] Hargrove LJ Simon AM Young AJ Robotic leg control with EMG decoding in an amputee with nerve transfers. N Engl J Med. 2013;369:1237–1242. 24066744 10.1056/NEJMoa1300126

[31] Kuiken TA Barlow AK Hargrove L Targeted muscle reinnervation for the upper and lower extremity. Tech Orthop. 2017;32:109–116. 28579692 10.1097/BTO.0000000000000194 PMC5448419

[32] Kung TA Langhals NB Martin DC Regenerative peripheral nerve interface viability and signal transduction with an implanted electrode. Plast Reconstr Surg. 2014;133:1380–1394. 24867721 10.1097/PRS.0000000000000168

[33] Irwin ZT Schroeder KE Vu PP Chronic recording of hand prosthesis control signals via a regenerative peripheral nerve interface in a Rhesus macaque. J Neural Eng. 2016;13:046007. 27247270 10.1088/1741-2560/13/4/046007

[34] Ursu DC Urbanchek MG Nedic A *In vivo* characterization of regenerative peripheral nerve interface function. J Neural Eng. 2016;13:026012. 26859115 10.1088/1741-2560/13/2/026012

[35] Srinivasan SS Carty MJ Calvaresi PW On prosthetic control: a regenerative agonist- antagonist myoneural interface. Sci Robot. 2017;2:eaan2971. 10.1126/scirobotics.aan2971 33157872

[36] Urbanchek MG Sando IC Irwin ZT Abstract: validation of regenerative peripheral nerve interfaces for control of a myoelectric hand by macaques and human. Plast Reconstr Surg Glob Open. 2016;4(9 Suppl);69–69.

[37] Chimutengwende-Gordon M Pendegrass C Blunn G The *in vivo* effect of a porous titanium alloy flange with hydroxyapatite, silver and fibronectin coatings on soft-tissue integration of intraosseous transcutaneous amputation prostheses. Bone Joint J. 2017;99-B:393–400. 28249981 10.1302/0301-620X.99B3.BJJ-2016-0360.R1 PMC5358203

[38] Allen MJ Houlton JE Adams SB The surgical anatomy of the stifle joint in sheep. Vet Surg. 1998;27:596–605. 9845224 10.1111/j.1532-950x.1998.tb00536.x

[39] Yoshida K Struijk JJ Horch KW Dhillon GS The theory of peripheral nerve recording. In: Neuroprosthetics: Theory and Practice. 2004 Singapore: World Scientific.

[40] Johnes and Other Wasting Diseases in Sheep. National Animal Disease Information Service. Available at http://www.nadis.org.uk/bulletins/johnes-and-other-wasting-diseases-in-sheep.aspx?altTemplate=PDF. Published 2014. Accessed June 29, 2014.

[41] Dowling RP To Develop Techniques that will Enhance Dermal Cell and Tissue Attachment in Order to Create a Seal and Prevent Infection of Implant Biomaterials used for ITAP. February 2015 Available at http://discovery.ucl.ac.uk/1461121/. Accessed June 29, 2018.

[42] Fitzpatrick N Smith TJ Pendegrass CJ Intraosseous transcutaneous amputation prosthesis (ITAP) for limb salvage in 4 dogs. Vet Surg. 2011;40:909–925. 22092391 10.1111/j.1532-950X.2011.00891.x

[43] Pantall A Hodson-Tole EF Gregor RJ Increased intensity and reduced frequency of EMG signals from feline self-reinnervated ankle extensors during walking do not normalize excessive lengthening. J Neurophysiol. 2016;115:2406–2420. 26912591 10.1152/jn.00565.2015 PMC4922462

[44] Cope TC Clark BD Motor-unit recruitment in self-reinnervated muscle. J Neurophysiol. 1993;70:1787–1796. 8294953 10.1152/jn.1993.70.5.1787

[45] Alvarez FJ Titus-Mitchell HE Bullinger KL Permanent central synaptic disconnection of proprioceptors after nerve injury and regeneration. I. Loss of VGLUT1/IA synapses on motoneurons. J Neurophysiol. 2011;106:2450–2470. 21832035 10.1152/jn.01095.2010 PMC3214094

[46] Bullinger KL Nardelli P Pinter MJ Permanent central synaptic disconnection of proprioceptors after nerve injury and regeneration. II. Loss of functional connectivity with motoneurons. J Neurophysiol. 2011;106:2471–2485. 21832030 10.1152/jn.01097.2010 PMC3214087

[47] Clites TR Carty MJ Ullauri JB Proprioception from a neurally controlled lower- extremity prosthesis. Sci Transl Med. 2018;10:eaap8373. 29848665 10.1126/scitranslmed.aap8373

[48] Clites TR Carty MJ Srinivasan S A murine model of a novel surgical architecture for proprioceptive muscle feedback and its potential application to control of advanced limb prostheses. J Neural Eng. 2017;14:036002. 28211795 10.1088/1741-2552/aa614b

[49] Hebert JS Olson JL Morhart MJ Novel targeted sensory reinnervation technique to restore functional hand sensation after transhumeral amputation. IEEE Trans Neural Syst Rehabil Eng. 2014;22:765–773. 24760915 10.1109/TNSRE.2013.2294907

[50] Hebert JS Chan KM Dawson MR Cutaneous sensory outcomes from three transhumeral targeted reinnervation cases. Prosthet Orthot Int. 2016;40:303–310. 26932982 10.1177/0309364616633919

[51] Loeb GE Gans C Electromyography for Experimentalists. 1986 Chicago: University of Chicago Press.

[52] Lowery MM Stoykov NS Taflove A A multiple-layer finite-element model of the surface EMG signal. IEEE Trans Biomed Eng. 2002;49:446–454. 12002176 10.1109/10.995683

[53] De Luca CJ Kuznetsov M Gilmore LD Inter-electrode spacing of surface EMG sensors: reduction of crosstalk contamination during voluntary contractions. J Biomech. 2012;45:555–561. 22169134 10.1016/j.jbiomech.2011.11.010

[54] van Vugt JP van Dijk J A convenient method to reduce crosstalk in surface EMG. Clin Neurophysiol. 2001;112:583–592. 11275529 10.1016/s1388-2457(01)00482-5

[55] Koh TJ Grabiner MD Evaluation of methods to minimize cross talk in surface electromyography. J Biomech. 1993;26(suppl 1):151–157. 8505349 10.1016/0021-9290(93)90086-t

[56] Lowery MM Stoykov NS Kuiken TA Independence of myoelectric control signals examined using a surface EMG model. IEEE Trans Biomed Eng. 2003;50:789–793. 12814247 10.1109/TBME.2003.812152

[57] Mentink MJA Taylor SJG Vanhoestenberghe A CAPITEL: design and implementation of a wireless 6 channel EMG measurement system for permanent in vivo use: in vitro results. 2017 In: International Functional Electrical Stimulation Society Conference London, UK; . Available at http://discovery.ucl.ac.uk/1566675/. Accessed June 22, 2018.

[58] de Jager K Mentink M Tjulkins F A multi- channel multiplexed EMG recording system to study electrode array configuration. 2018 In: Proceedings of BioMedEng18 London, UK.

[59] Lancashire HT Implantable Electrodes for Upper Limb Prosthetic Control. September 2015 Available at http://discovery.ucl.ac.uk/1470872/. Accessed November 25, 2015.

