# Supplementary material for: Hard-wired Epimysial Recordings from Normal and Reinnervated Muscle Using a Bone-anchored Device
Source: Plast Reconstr Surg Glob Open. 2019 Sep 23;7(9):e2391. doi: 10.1097/GOX.0000000000002391 (PMC6799399; doi:10.1097/GOX.0000000000002391)
Supplement: Supplementary file 1 [file gox-7-e2391-s001.pdf]

Supplemental Digital Content

Hard-wired Epimysial Recordings from Normal and Reinnervated Muscle using a Bone-Anchored Device

Lancashire HT, Al Ajam Y, Dowling RP, Pendegrass CJ, Blunn GW.

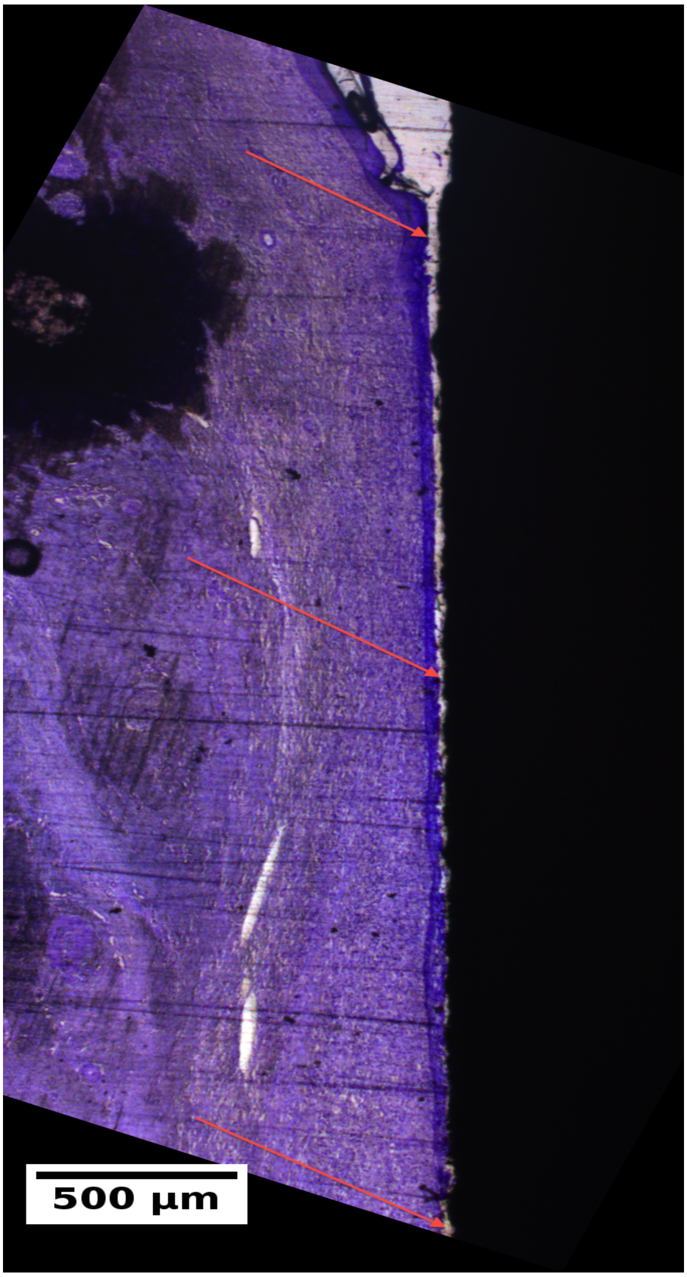

Figure Supplemental Digital Content 1: Light micrograph of the skin-implant interface stained with Toluidine blue. Epidermal downgrowth and sinus track formation along the implant shaft is visible (arrows). Rotated and cropped to show vertical implant edge on right. Modified with permission [41].
