# Supplementary material for: Hard-wired Epimysial Recordings from Normal and Reinnervated Muscle Using a Bone-anchored Device
Source: Plast Reconstr Surg Glob Open. 2019 Sep 23;7(9):e2391. doi: 10.1097/GOX.0000000000002391 (PMC6799399; doi:10.1097/GOX.0000000000002391)
Supplement: Supplementary file 2 [file gox-7-e2391-s002.pdf]

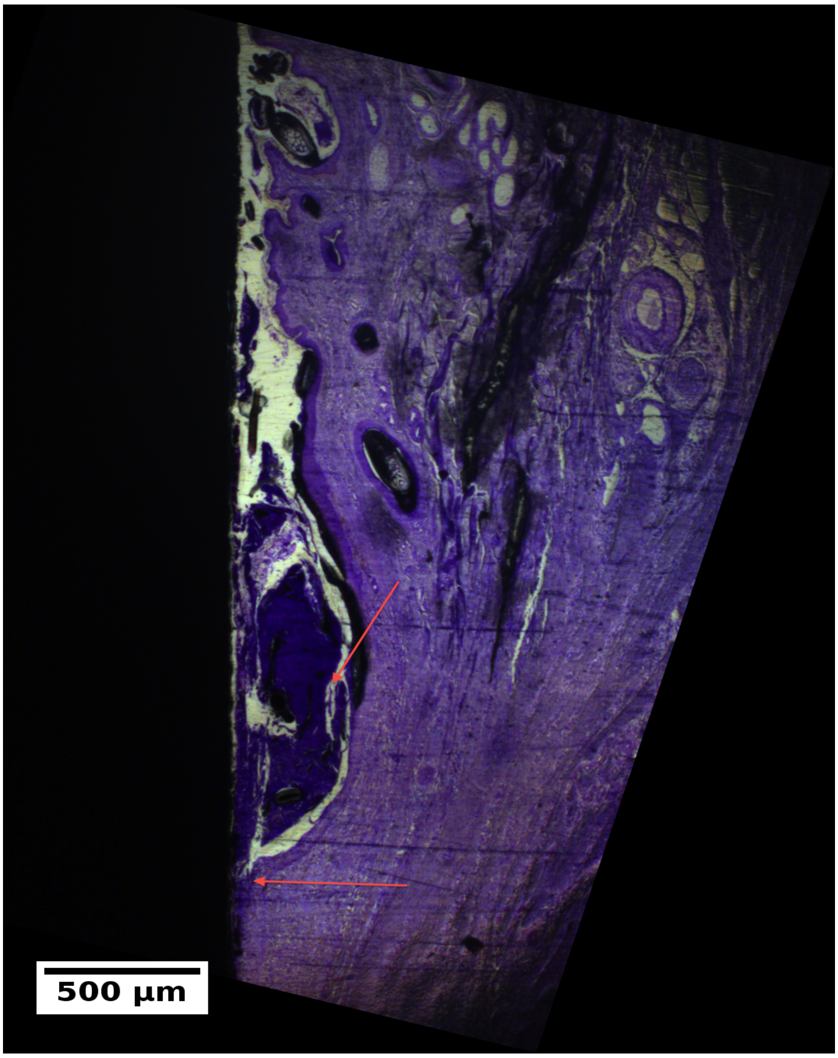

Figure Supplemental Digital Content 2: Light micrograph of the skin-implant interface stained with Toluidine blue. Debris impeding epidermal attachment is visible at the base of a sinus. Epidermal attachment to occurs immediately below the debris. Rotated and cropped to show vertical implant edge on left. Modified with permission [41].
