# Supplementary material for: Hard-wired Epimysial Recordings from Normal and Reinnervated Muscle Using a Bone-anchored Device
Source: Plast Reconstr Surg Glob Open. 2019 Sep 23;7(9):e2391. doi: 10.1097/GOX.0000000000002391 (PMC6799399; doi:10.1097/GOX.0000000000002391)
Supplement: Supplementary file 4 [file gox-7-e2391-s004.pdf]

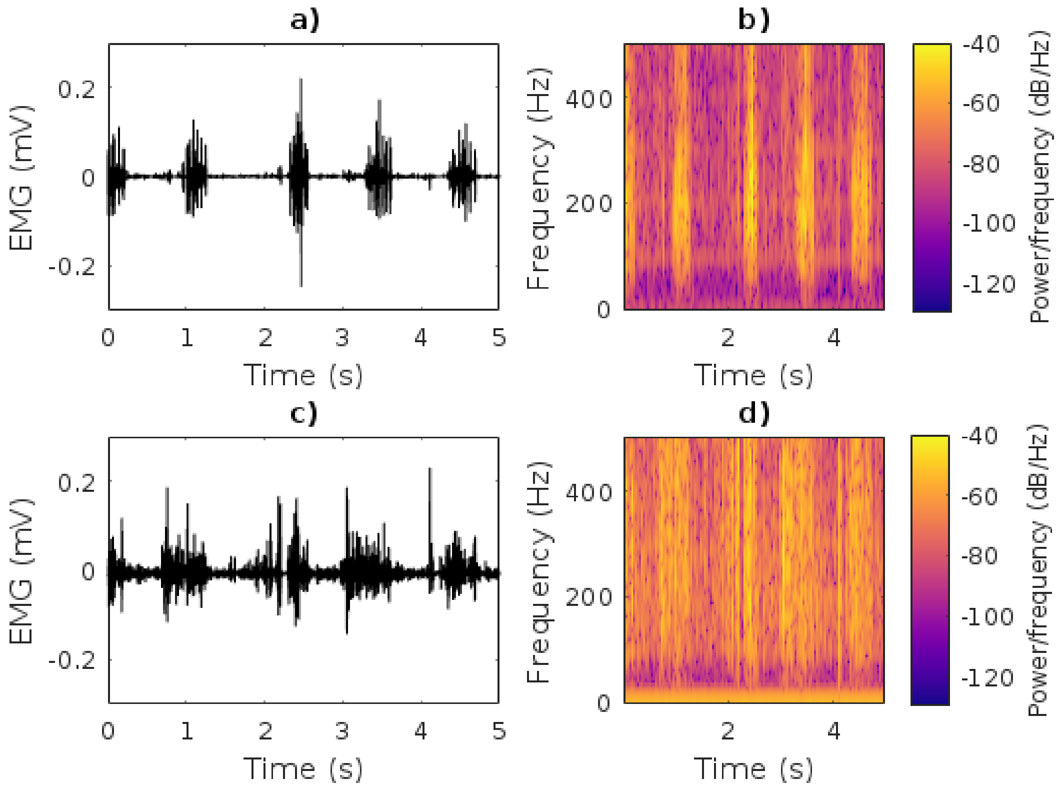

Figure Supplemental Digital Content 4: Raw EMG (a,c) and power spectrogram (b,d) of EMG recordings from epimysial electrodes (a,b) and skin surface electrodes (c,d) after 19 weeks. Example traces recorded coincidentally from the same animal. Spectrograms by Fourier transform with 50 sample Hamming windows.
