# Supplementary material for: Hard-wired Epimysial Recordings from Normal and Reinnervated Muscle Using a Bone-anchored Device
Source: Plast Reconstr Surg Glob Open. 2019 Sep 23;7(9):e2391. doi: 10.1097/GOX.0000000000002391 (PMC6799399; doi:10.1097/GOX.0000000000002391)
Supplement: Supplementary file 5 [file gox-7-e2391-s005.pdf]

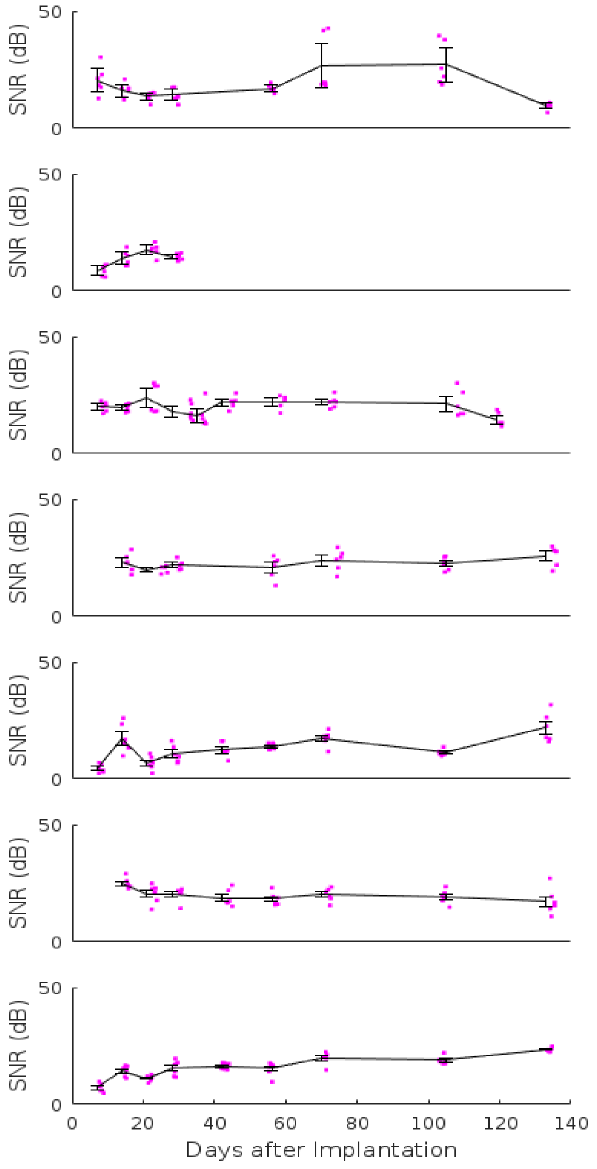

Figure Supplemental Digital Content 5: Summarised signal-to-noise ratio data for each individual animal with the standard surgical procedure. n=7 animals of which data for 5 animals to 19 weeks. Data plotted as means ± 95% Confidence Intervals. Data summarised in Figure 4(a).
