# Supplementary material for: Hard-wired Epimysial Recordings from Normal and Reinnervated Muscle Using a Bone-anchored Device
Source: Plast Reconstr Surg Glob Open. 2019 Sep 23;7(9):e2391. doi: 10.1097/GOX.0000000000002391 (PMC6799399; doi:10.1097/GOX.0000000000002391)
Supplement: Supplementary file 6 [file gox-7-e2391-s006.pdf]

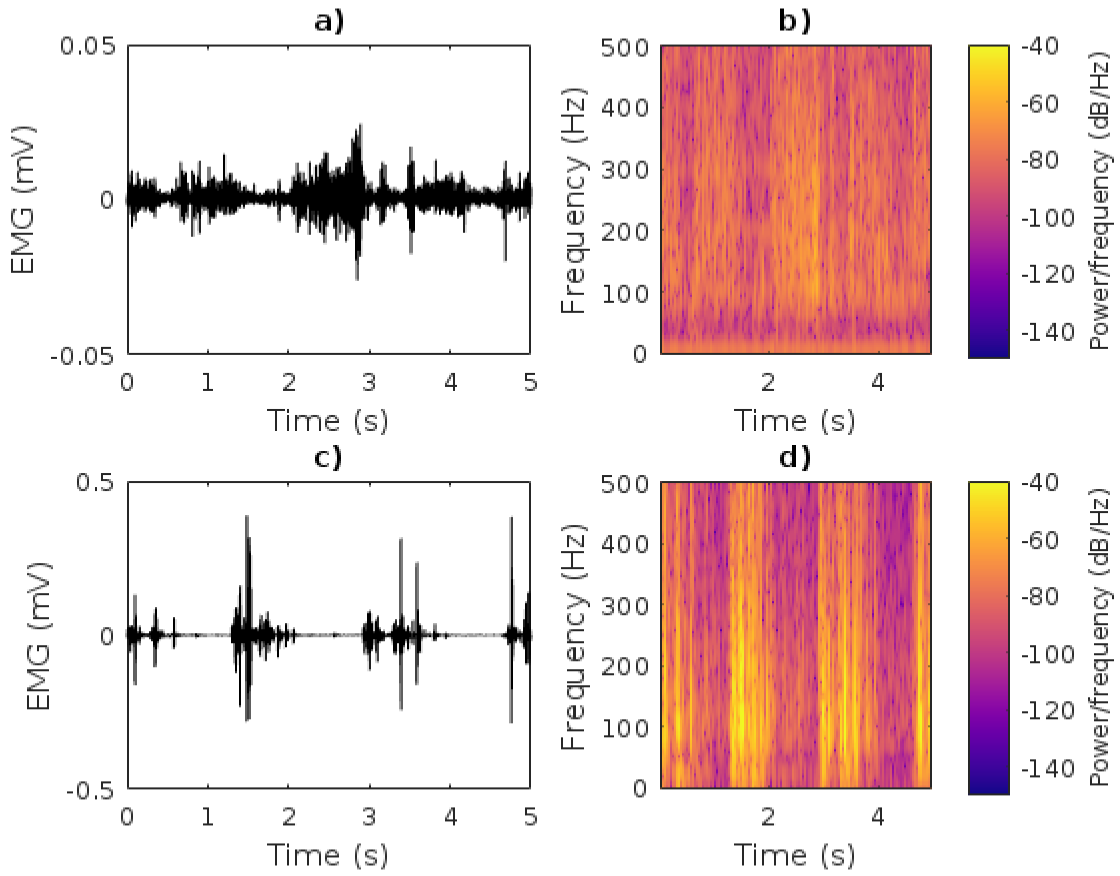

Figure Supplemental Digital Content 6: Raw EMG (a,c) and power spectrogram (b,d) of EMG recordings following targeted muscle reinnervation. Recordings made at: a,b) 3 weeks; c,d) 10 weeks. Note the change in scale for the EMG plots. Spectrograms by Fourier transform with 50 sample Hamming windows.
